# A 10-year observation of PM_2.5_-bound nickel in Xi’an, China: Effects of source control on its trend and associated health risks

**DOI:** 10.1038/srep41132

**Published:** 2017-01-24

**Authors:** Hongmei Xu, Steven Sai Hang Ho, Junji Cao, Benjamin Guinot, Haidong Kan, Zhenxing Shen, Kin Fai Ho, Suixin Liu, Zhuzi Zhao, Jianjun Li, Ningning Zhang, Chongshu Zhu, Qian Zhang, Rujin Huang

**Affiliations:** 1 Department of Environmental Science and Engineering, Xi’an Jiaotong University, Xi’an, China; 2 Key Lab of Aerosol Chemistry & Physics, Institute of Earth Environment, Chinese Academy of Sciences, Xi’an, China; 3 Laboratoire d’Aérologie, Université de Toulouse, CNRS, UPS, France; 4 Division of Atmospheric Sciences, Desert Research Institute, Reno, Nevada, USA; 5 Institute of Global Environmental Change, Xi’an Jiaotong University, Xi’an, China; 6 School of Public Health, Key Lab of Public Health Safety of the Ministry of Education, Fudan University, Shanghai, China; 7 School of Public Health and Primary Care, The Chinese University of Hong Kong, Hong Kong, China

## Abstract

This study presents the first long term (10-year period, 2004–2013) datasets of PM_2.5_-bound nickel (Ni) concentration obtained from the daily sample in urban of Xi’an, Northwestern China. The Ni concentration trend, pollution sources, and the potential health risks associated to Ni were investigated. The Ni concentrations increased from 2004 to 2008, but then decreased due to coal consumption reduction, energy structure reconstruction, tighter emission rules and the improvement of the industrial and motor vehicle waste control techniques. With the comparison of distributions between workday and non-workday periods, the effectiveness of local and regional air pollution control policies and contributions of hypothetical Ni sources (industrial and automobile exhausts) were evaluated, demonstrating the health benefits to the populations during the ten years. Mean Ni cancer risk was higher than the threshold value of 10^−6^, suggesting that carcinogenic Ni still was a concern to the residents. Our findings conclude that there are still needs to establish more strict strategies and guidelines for atmospheric Ni in our living area, assisting to balance the relationship between economic growth and environmental conservation in China.

Nickel (Ni) is one of the trace metals widely-spread in atmospheric environment^1^. It is known to be released from natural source and anthropogenic activities with either stationary or mobile inputs^2^,^3^. The natural sources of Ni in particulate matter (PM) include soil dust, volcanic emission, forest fire, vegetation, sea salt and meteoric dust^3^,^4^. The anthropogenic inputs are dominated by combustion of diesel, fuel oil and coal, high temperature metallurgical operations, and miscellaneous^4^. Ni can be also found in emissions from environmental tobacco smoke, stainless steel kitchen utensils and home heating and cooking^5^,^6^. Ambient levels of Ni vary considerably but the highest values were often reported in highly industrialized areas^3^, in which Ni is used for the production of stainless steel and other nickel alloys.

Coal combustion is the predominant source of anthropogenic atmospheric Ni, with 63.4% of the national total Ni emission in China^6^. Liquid fuels consumption and biofuel are the next two important ones, contributing to 12.4% and 8.4% of the total emissions. Other studies showed that Ni is typically associated with the combustion of heavy fuel oil^7^, while more than 80% Ni in the air is estimated due to exhaust emissions^8^. In Xi’an, the major source of Ni in fine particulate matter (PM_2.5_, PM with aerodynamic diameters ≤2.5 μm) is fossil fuel combustion^9^. With an increase of fossil fuels consumption and industrial production in China, it is valuable to characterize Ni levels over time and assess its current trend.

Ni is designated as an acute toxic substance by many governmental agencies and international institutes and is classified as a human carcinogen (Group 2B) by the International Agency for the Research on Cancer (IARC)^10^ and the World Health Organization (WHO)^11^. Ni can harm various organisms and its toxicity varies considerably depending on its speciation, physical form, concentration level and exposure pathway^1^. Generally, the soluble Ni-compounds are more toxic than the less-soluble ones. Nickel tetracarbonyl, Ni(CO)_4_, has been found to be the most toxic, as reported the influences on industrial workers^12^. Exposure to Ni and its associated compounds can cause a variety of adverse effects on human health^13^,^14^, among which the most important are developmental, genotoxic, neurological, reproductive, and carcinogenic^12^. Using the National Morbidity, Mortality, and Air Pollution Study database, Lippmann *et al*.^15^ found that daily mortality rates in the 60 United States cities with speciation data were significantly associated with average levels of Ni and V. Therefore, it is necessary to investigate the characteristics and health risks of atmospheric Ni in rapid developing regions over a long term period.

This research has been conducted as part of a 10-year monitoring of daily PM_2.5_ in Xi’an, one of the most polluted cities in the world^16^,^17^, aimed to expand our knowledge base regarding to the temporal distribution, source characteristics and health risks of Ni in PM_2.5_. Owing to these purposes, the concentrations of airborne Ni were measured in an urban environment of Xi’an during the period 2004–2013. We aimed at (i) investigating the contributions of industrial sources (including fuel oil and coal combustions) and motor vehicle emissions to the Ni pollution in this typical urban area, and (ii) assessing the effects of the control of such emissions on Ni concentrations and related health risks.

## Results and Discussion

### Temporal variation of Ni in PM_2.5_ over ten years

A summary of airborne PM_2.5_ mass and Ni concentrations for a 10-year period (2004–2013) is presented in Table 1. The daily PM_2.5_ concentrations ranged from 7.6 to 778.8 μg m^−3^, with an average of 169.0 ± 101.2 μg m^−3^. The annual average concentrations of PM_2.5_ had the highest of 197.8 ± 107.7 μg m^−3^ in 2006 and the lowest of 145.7 ± 76.4 μg m^−3^ in 2011, showing a slight decline with fluctuations in the recent years. The daily Ni concentrations ranged from <0.08 (i.e., the Minimum Detection Limit (MDL) for Ni) to 88.1 ng m^−3^ (Figure S1 in Supplementary Information (SI)), while the annual average values dropped from 7.7 ± 8.3 ng m^−3^ in 2007 to 3.0 ± 2.6 ng m^−3^ in 2013, with a consistent trend with the PM_2.5_ mass.

The 10-year average Ni concentration (5.9 ± 5.5 ng m^−3^) was well below the recommended level of 25 and 20 ng m^−3^ defined by WHO^12^ and European Union (EU)^18^, respectively. There is no Ni limit established in National Ambient Air Quality Standards (NAAQS) in China^19^. The emission standard of Ni and its related compounds for copper, nickel and cobalt industry during smelting processes restricts that their emissions must not exceed 4.3, 4.3, and 0.04 mg m^−3^ for existing facility, new facility, and enterprise boundary, respectively, in China^20^. Such limits were only effective on enterprises and industry sector. Therefore, the recommendation Ni levels in the regional and worldwide guidelines are always too high, leading to overlook its risk in China.

In our previous research^21^ based on the same samples, the PM components, organic carbon (OC), elemental carbon (EC), 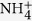, Cl^−^, 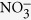, Cl, and Ni, showed the strongest positive associations and were statistically significant in Xi’an. Ni was strongly associated with all three mortality outcomes for at least 1-day lagged exposure. The Inter Quartile Range increase of 0.01 μg m^−3^ in 1-day lagged Ni was associated with 0.4% (95% confidence interval (CI): 0.0%, 0.8%), 0.6% (95% CI: −0.1%, 1.2%), and 0.9% (95% CI: 0.2%, 1.7%) increases in total, cardiovascular, and respiratory mortality, respectively. Although the 10-year average Ni concentration was well below the limits mentioned above, the negative health effects of Ni are obvious.

The comparison of the Ni concentrations in this study with latest 15-years data available in China^22^ shows that the Ni concentration in Xi’an was lower than those in Beijing (20.0 ng m^−3^ in 2005–2006) and Chongqing (30.0 ng m^−3^ in 2005–2006)^23^, but higher than the values measured in Chengdu (3.7 ng m^−3^ for 2009 spring)^24^ and Fuzhou (4.2 ng m^−3^ for 2007–2008)^25^, and comparable with the data obtained in Shanghai (10.0 ng m^−3^ for 2004–2005)^26^ and Hong Kong (5.9 ng m^−3^ for 2000 winter)^27^. It was comparable with that reported in Korea, falling in the range of 3.7–12.6 ng m^−3^ from 1998 to 2010^1^.

**Figure 1:**
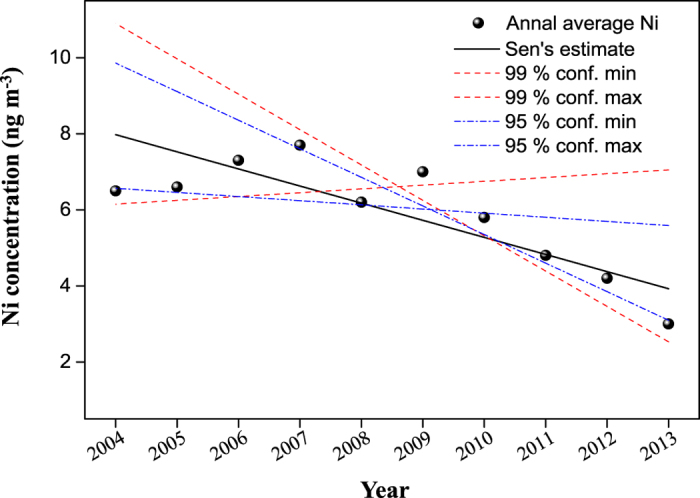
Sen’s estimation with Mann-Kendall statistics for annual concentrations of Ni from 2004 to 2013.

The non-parametric Sen’s slope estimation method and the non-parametric Mann-Kendall test were applied to test the presence of monotonic trend of the Ni concentration (either increase or decrease) and estimate the slope of a linear trend over the entire study period^28^. The Mann-Kendall test showed an apparent decline trend of −0.45 ng m^−3^ year^−1^ (Q) with statistical significance at a confidence level of *p* < 0.01 (Fig. 1). This suggests that Ni-related PM_2.5_ pollution control efforts by the local government have resulted in measurable positive effects to the environment. Moreover, by inter-comparing the data obtained in Xi’an, Zhang *et al*.^29^ showed the annual average Ni concentration of 510 ng m^−3^ in total suspended particles (TSP) in 1997, and 180 ng m^−3^ in PM_10_ (PM with aerodynamic diameters ≤10 μm) in 1998, were 86 and 30 times higher than the Ni level measured in this study. It hence is a further evidence that a huge improvement of Ni pollution has been achieved in this city.

The whole monitoring duration could be divided into two phases in light of the distinctive characteristics in the Ni concentration trend (Fig. 1). In the first phase (from 2004–2007, designated as Phase I), the annual average concentrations increased with a rate of 6.2% per year. In contrast, the second phase (from 2009–2013, named as Phase II), displayed a distinct decreasing trend at an annual change of −10.3%. The year of 2008 is the turning point among the ten years, when the Ni concentration began to drop down. Figure S2 in SI compares the daily and monthly variations of Ni concentrations among 2007, 2008 and 2009 in Xi’an. It is clear that the Ni concentrations were significantly lower in January, February, and May to September of 2008, in comparison with the same periods in 2007 and 2009. The low Ni values in January and February, 2008 could be influenced by a consequence of a persistent low-temperature event over a large region of China (including Xi’an) from January 15^th^ to February 10^th^, 2008, resulting in freezing rain and snow^30^,^31^ and possibly leading the air dilution in the atmosphere.

In addition, the lower Ni levels in May to September of 2008 could be strongly related to the 29^th^ Olympics Games held in Beijing in 2008. The Beijing Municipal Government and peripheral provinces authorities have implemented various environmental control measures to reduce air pollutants emissions^32^, directly contributing to the improvement of Beijing-Tianjin-Hebei region atmospheric environment and indirectly promoting the release a series of air pollution control policies and practical actions in surrounding provinces and cities (including Xi’an). Under the guide of the “Green Olympics”, the existing air pollution control policies and relatively strict law enforced, leading to improve atmospheric environment in Xi’an. Moreover, it is worth mentioning that Xi’an is located in downwind position of Northern China (particularly of Shanxi, Hebei and Beijing). While air quality in Northern region was better, less regional transportation of pollutants was approached to Xi’an.

Comparing the large difference of Ni concentrations between Phase I (2004–2007) and Phase II (2009–2013), the increase of Ni concentrations in Phase I was evident, mainly caused by rapid economic development, increases in consumption of coal (Figure S3A in SI, from 2004 to 2007) and fuel oil burning, and speedy growth in numbers of motor vehicle (Table S1 in SI)^33^. During the Phase II, significantly drop in Ni concentration was resulted from the coal consumption reduction (2009–2012) and energy structure reconstruction (i.e., the unitization of coal as energy had been reduced from 2008 to 2013) (Figure S3A in SI)^33^. Both of the reinforcement of environmental laws and regulations, implementation of the exhaust gas purification equipment, increased investment in waste gas treatment and disposal capacity (Figure S3B in SI) are key factors as well^33^.

The variations of Ni/PM_2.5_ ratios were different from those of PM_2.5_ mass concentrations (Table 1). The minimum annual average PM_2.5_ was observed in 2011, preceding an increase in 2012–2013. These were consistent with the rapid growth of economy, high energy consumption, the expansion of civil vehicles, and the increase of construction activities associated to the real estate industry boom in Xi’an after 2011^33^,^34^. In addition, there were unfavorable meteorological conditions leading to severe haze events in the winters of 2011–2013^35^. The Ni/PM_2.5_ ratio reached a maximum of 0.043% in 2009 then decreased, suggesting that Ni levels have been successfully lowered by the enhanced control of air pollution emissions.

Seasonal and monthly variations of Ni concentrations displayed no obvious pattern over the studied period (Figures S4 and S5 in SI) in Xi’an. The seasonal characteristics varied from one year to the other, possibly due to the complexity and changeability of the local or regional pollution sources, which include soil dust and combustion processes related to heavy industrial operations and traffic. On average, the seasonal Ni concentrations had the descending following order: winter (6.9 ng m^−3^) > spring (6.7 ng m^−3^) > autumn (5.7 ng m^−3^) > summer (4.6 ng m^−3^), in consistence with the observations from previous study^36^. Additionally, the highest Ni concentrations in May were observed in this study^37^. Unfortunately, we could not find any emission data on seasonal or monthly waste gas from local industries in Xi’an. The reason of the highest Ni concentration in May needs further study and investigation.

### Ni concentration between workday and non-workday periods

Based on the main sources of Ni, these is a hypothesis: Ni emission sources have a very strong relationship with the factory work arrangement (work-rest schedule) and the traffic pattern (rush versus non-rush hours and workday versus non-workday). In order to verify this hypothesis, the comparison of long-term distributions of Ni concentrations were examined between workday and non-workday periods. Moreover, the impacts from two critical pollution sources of Ni, including the industrial activities and traffic frequency, were thus identified to evaluate the PM_2.5_-bound Ni pollution control policies effectiveness during the ten years.

**Figure 2:**
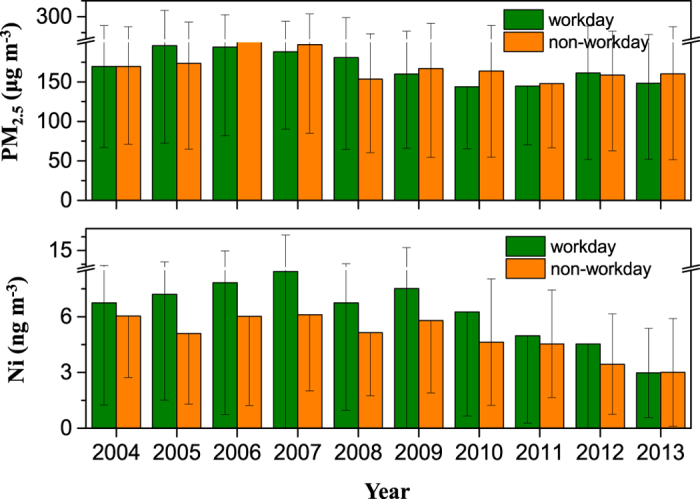
A comparison of PM_2.5_ and Ni concentrations between the workday and non-workday periods over ten years.

Table S2 in SI summarizes the Chinese statutory holidays between the study years. The average annual PM_2.5_ mass concentrations on the working and non-working days were 168.7 ± 20.1 and 170.8 ± 18.1 μg m^−3^, respectively, and the average annual concentrations of Ni were 6.3 ± 1.7 and 5.0 ± 1.1 ng m^−3^, respectively, over the 10-year period (Fig. 2). Only a little difference was seen on the PM_2.5_ levels. Its workday to non-workday ratio (W/N) was close to 1.0, suggesting that a complex pollution mix of PM predominate any simple source pattern driven by the regular working pattern. As one can expect, higher Ni concentrations were seen in the workdays. The annual W/N ratios of Ni averaged 1.3, which can be attributed to two potential causes: (1) the factories shuttered or reduced their discharge; and (2) the demands of motor vehicle transportations (i.e., almost no traffic jam was seen in morning-evening rush hours) were subdued during the non-workday period.

It should be noted that the Ni concentrations in the workday and non-workday had distinguishable differences between the two phases. Regarding workdays, the Ni concentrations continuously increased during Phase I (2004–2007), then decreased during Phase II (2009–2013), which dominated the Ni trend during the ten years. This phenomenon might be resulting from the drastic decrease on industrial and motor vehicle emission rates after 2009, despite the continued growth in economy together with the enhanced consumptions of raw coal and crude oil and the increasing possession of civil vehicles (Table S1 in SI). In Xi’an, the raw coal consumption increased by 96% from 5.2 million ton in 2004 to 10.3 million ton in 2013, the crude oil usage increased by 50% from 1.4 million ton in 2004 to 2.1 million ton in 2013, and the possession of civil vehicles also increased by 263% from 0.5 million unit in 2004 to 1.9 million unit in 2013^33^.

Moreover, the energy structure reconstruction, especially the decline of coal consumption, in China was the major reason for the decrease of atmospheric Ni level from the year of 2010. The proportion of coal used as energy in 2013 was 66.0%, which was 7.2% lower than that in 2007 (71.1%) (Figure S3A in SI), while the percentage of crude oil consumption had been declined by 13.6% from 2004 to 2013. Natural gas and other new energy sources, such as hydro, nuclear and wind powers had been increased by 132% and 46.3% from 2004 to 2013. Such reconstruction of energy structure could be the most important factor for the reduction of Ni in Xi’an^33^.

The lower Ni concentrations in the recent years could be ascribed to the nationwide implementation of air pollution control strategies for the plants and factories, including power plant scrubbers, electrostatic precipitators, waste-heat recovery systems, smelting scrubbers, and scrubbers for industrial boilers and inert-gas generators^38^. As mentioned above, increased investment and improved disposal capacity in treatment of waste gas led to purify the exhaust of raw coal, crude oil, and automobile fuels, resulting in a decline on Ni level in the atmosphere (Figure S3B in SI). However, the less decline of Ni concentrations in the non-workday period represents that the sources during the period were relatively stable during the ten years and the dominated contributions to Ni for workday were from industrial activities and traffic frequency.

### Ni sources implication by using enrichment factor

The 10-year average enrichment factor (EF) of Ni was 7.3 ± 2.7. Compared to the crust-derived elements, this EF value for Ni is more than 5 times higher, which further underlines the predominant influence mainly from anthropogenic sources in Xi’an^39^. In our study, the annual average EFs ranged from 3.8 ± 1.3 in 2013 to 10.2 ± 4.6 in 2005 (Table 2). The fitting linear regression equation of Ni EF values had a negative slope, suggesting a decrease of the share of anthropogenic activities contributed to the atmospheric Ni levels in the recent years.

The highest EF was often seen in summer (9.5 ± 3.6 in Table 2), pointing out a relatively high contribution from anthropogenic sources due to slight fugitive dust with more precipitation and higher relative humidity^40^. The lowest Ni EF values were observed in March and April (<5) (in spring, Table 2 and Figure S6 in SI), suggesting that Ni in spring was mainly affected by the natural sources, especially during the dust episodes, underlining that soil dust was the major source in spring^41^. Elevated Ni concentrations and relatively high EFs in winter might be attributed to the increases of residential coal burning and a stable atmospheric boundary layer during the heating-supply period in Xi’an (15^th^ November to 15^th^ March).

**Figure 3:**
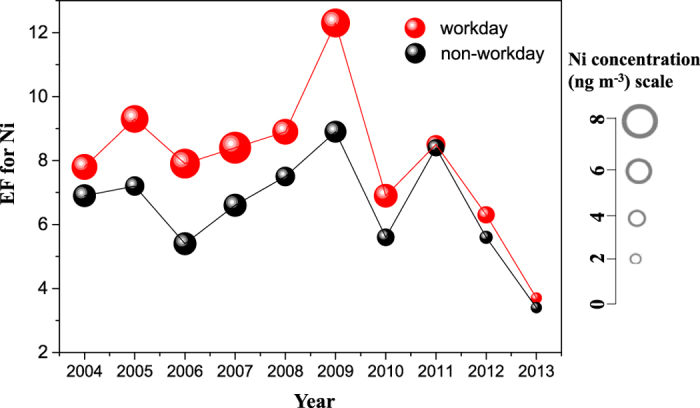
Variations of enrichment factors (EFs) of Ni in PM_2.5_ between the workday and non-workday periods (using Fe as the normalizing element; spot sizes represent the concentrations of Ni).

The average EFs of Ni were 7.8 ± 2.2 (ranging from 3.7 to 12.3) and 6.4 ± 1.6 (ranging from of 3.4 to 8.9) respectively, in the workday and non-workday periods, respectively (Fig. 3). Their trends were basically similar among the years. The higher EF in the workdays was more inclined to derive from non-crustal sources, i.e., anthropogenic emissions, in consistence with the higher Ni concentrations discussed in previous section. From the years 2004 to 2009, the average W/N ratio of Ni EF was 1.3, which was higher than the average ratio of 1.1 that of measured in 2010–2013, representing a lessening of non-working effect since 2009.

### Health risk assessment: Non-cancer and cancer risks of Ni

**Figure 4:**
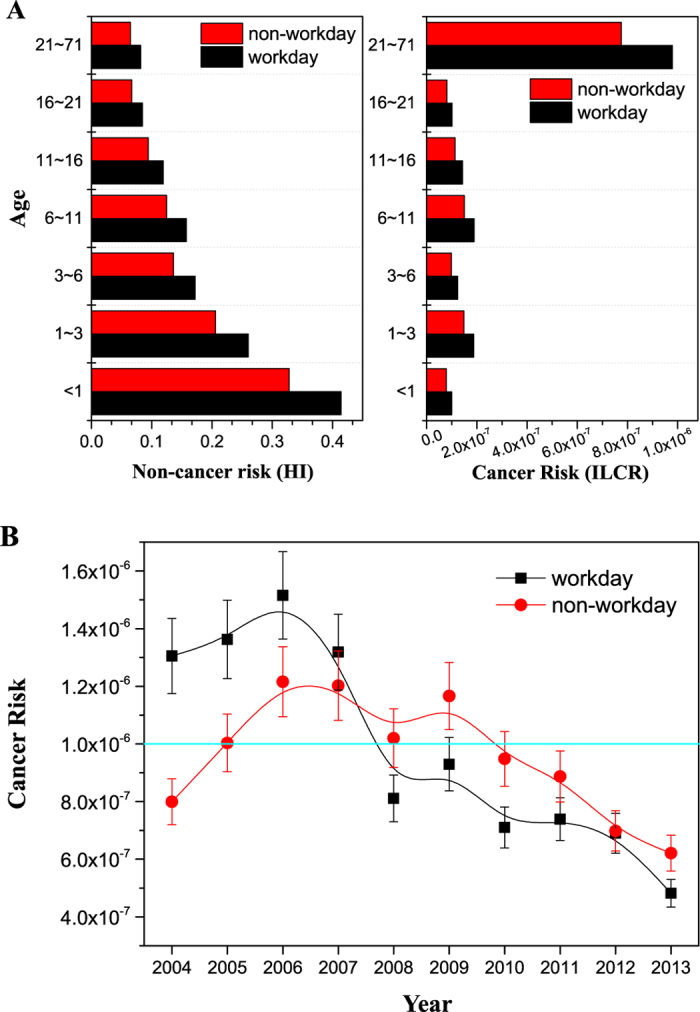
(**A**) Comparison of non-cancer (HI) and cancer (ILCR) risks of Ni at different age groups and for the workday and non-workday in Xi’an. (**B**) Annual average cancer risk (ILCR) values of Ni in PM_2.5_ for workday and non-workday in the period of 2004–2013 (light blue line represents the threshold value of ILCR; error bars represent standard deviations of ILCR values).

Both inhalational non-cancer hazard index (HI) and incremental lifetime cancer risk (ILCR) of Ni were assessed for Xi’an residents (Fig. 4A). We divided the population into seven age groups and classified exposure duration into workday and non-workday periods.

Most trace metals could be retained in body for a long period of time and potentially cause serious non-cancer adverse effects to human^42^. The HI values were all well below 1.0 (0.39 and 0.08 for <1 year old children and 21–71 years old adults, respectively). However, higher HI values were obtained for small aged group in both workday and non-workday, indicating that children were more sensitive to non-carcinogenic effects and should hence minimize their exposures to Ni^43^. For all ages, the average total HI was 0.15, representing no adverse health effects to the population in Xi’an. The HI of Ni in the workday (an average of 0.16) was 1.3 times of that in the non-workday (an average of 0.12).

The ILCR value was 1.1 × 10^−6^ for all ages (95% CI for Ni average concentration) in both workday and non-workday periods, which was higher than the threshold value of 10^−6^, demonstrating that carcinogenic Ni was a concern to the residents and its emission should be controlled effectively in Xi’an. The ILCR for adults was higher than that for children, while the value in the workday (1.1 × 10^−6^) was higher than that in the non-workday (9.0 × 10^−7^).

The variations of Ni ILCR via inhalation over the ten years are shown in Fig. 4B. The ILCR values ranged from 4.8 × 10^−7^ to 1.5 × 10^−6^ (an average of 1.0 × 10^−6^) and from 6.2 × 10^−7^ to 1.2 × 10^−6^ (an average of 9.5 × 10^−7^) in the workday and non-workday periods, respectively. An obvious decline was observable from 2006 due to the gradual implementation of stricter air pollution control policies. The ILCR of Ni was above 10^−6^ for workdays from 2004 to 2007, and for non-workdays from 2005 to 2009. For the workdays, the maximum ILCR was more than 3 times of its minimum value, while it was only 1.9 times for the non-workdays. The net decrease of the Ni cancer risk for workdays confirmed the importance and effectiveness of the control measures applied to industries and traffic. From 2010, the ILCR of Ni fell under the threshold value, indicating a negligible cancer risk to the residents in Xi’an either in the working or non-working periods. The implementations of motor vehicle and industrial emissions control policies, reconstruction of energy structure and advanced technologies have led to a benefit to human health seen in our long-term monitoring.

### Uncertainty analysis of health risk assessment

Uncertainty arose from Ni exposure, risk estimation and other effects. In order to quantify the uncertainty of the estimated risks, a Monte Carlo simulation was implemented to find out a more realistic view on the risk distribution in this study^44^. We have performed independent runs at 3000, 5000 and 10000 iterations with each parameter sampled independently from the appropriate distribution at the start to test the convergence and the stability of the numerical output^45^. The result showed that the 5000 iterations were sufficient to ensure the stability of results. A confidence interval for estimated risk was determined on the basis of the 5^th^ and 95^th^ quartiles of the simulation outcomes. The Monte Carlo simulation was implemented by using Crystal Ball software (Version 11.1.2.2, Decisioneering, Inc., CO, USA). The probability and cumulative probability distributions of the calculated ILCRs for the workday and non-workday in Xi’an are presented in Figure S7 in SI.

The normal distributions of Ni ILCRs for residents did not show statistical skew (Figure S7 in SI), displaying that the inhalation ILCR in the workday was higher than that in the non-workday. The median values, the 5^th^ and 95^th^ percentile of ILCRs were estimated to be 1.13 × 10^−6^, 0.88 × 10^−6^, 1.42 × 10^−6^ and 0.90 × 10^−6^, 0.70 × 10^−6^, 1.13 × 10^−6^ in the workday and non-workday periods, respectively. Our results indicated that 80.4% (100% minus 19.6%) of the workday and 21.9% (100% minus 78.1%) of the non-workday inhalation ILCRs of Ni were equal or greater than 10^−6^, indicating a higher potential carcinogenic risk in the workday period. Even though the Ni concentrations decreased in recent years, it is still necessary to take more appropriate measures to control the exposure to Ni in Xi’an.

We acknowledged that some limitations existed in this study. The non-cancer and cancer risks estimations were potentially underestimated due to only single element was focused. However, Ni should be the closest correlated trace element with health in PM_2.5_ as shown in the previous study^21^. Besides, the estimations of health risks also require more accurate individual exposure data and possible synergistic effects with other heavy metals (such as Pb and Cr) and factors. Further investigation on potential health effects with hospital health data and detailed emission data is highly recommended.

## Methods

### Sample collection

The sampling site was located in the southeastern part of downtown Xi’an, China, where stands the Xi’an hi-tech industries development zone. This zone consisted in four pillar industries, including (i) electronic information, (ii) advanced manufacturing, (iii) bio-medicine, and (iv) modern service industry, with a mixture of industrial, commercial, residential, and traffic environments^46^,^47^. It is worth emphasizing that a battery manufacturer (Ni related battery) and an automobile plant (welding of metals) were located within less than 7 km from the sampling site.

24-hour integrated daily PM_2.5_ samples were collected (from 10:00 am to 10:00 am on the next day, local time) from 1^st^ January 2004 to 31^th^ December 2013. A total of 3,534 valid samples were collected during the study period. The PM_2.5_ samples were collected on pre-fired (780 °C, 3 hours) 47 mm Whatman quartz microfibre filters (QM/A^®^, Whatman Inc., U.K.) with the mini-volume air samplers (Airmetrics, Eugene, OR, USA) that operated at a flow rate of 5 L min^−1^. The samplers were deployed ~10 m above ground level, on the roof of a two-story building of Institute of Earth Environment, Chinese Academy of Sciences (IEECAS) (34°13′N, 108°52′E). The samples were categorized into four seasons based on the local meteorological conditions in Xi’an (Spring: March-May; Summer: June-August; Autumn: September-November, and Winter: December-February). All exposed filters were placed in clean plastic air-tightly sealed bags and returned to the laboratory. The filters were stored in a refrigerator at <4 °C before analysis to minimize the evaporation of volatile components. Besides, Table S3 in SI shows the meteorological factors in Xi’an from 2004 to 2013^33^.

### Mass analysis

The PM_2.5_ mass concentrations were obtained by weighing the filters with a Sartorius ME 5-F electronic microbalance that had a sensitivity of ±1 μg (Sartorius, Gottingen, Germany) after equilibration at a temperature of 20 °C–23 °C and a relative humidity (RH) of 35–45% for at least 24 hours. Each sample was weighed at least two times before and after sampling. The allowable absolute errors between duplicate weights were set to ≤0.015 mg for blank filters and ≤0.020 mg for loaded samples.

### Ni analysis and QA/QC

Energy Dispersive X-Ray Fluorescence (ED-XRF) spectrometry (Epsilon 5 ED-XRF analyzer, PANalytical, Netherlands) was used to determine the Ni concentrations on the quartz fiber PM_2.5_ filters^48^,^49^,^50^. The X-ray source is a side-window X-ray tube with a gadolinium anode, and it is operated at an accelerating voltage of 25 to 100 kV and a current of 0.5 to 24 mA (maximum power: 600 W). The characteristic of X-ray radiation is detected by a germanium detector (PAN 32). In this study, the ED-XRF spectrometer was calibrated with thin-film standards obtained from MicroMatter Co. (Vancouver, Canada).

National Institute of Standards and Technology (NIST) Standard Reference Material (SRM) 2783 was employed to validate the accuracy of Ni measurement. The reference value for Ni in SRM 2783 is 68 ± 12 ng filter^−1^, which was similar to the results analyzed by our ED-XRF system: the average concentration of Ni was 63 ± 14 ng filter^−1^ with seven replicate analyses. The relative error was 7.3% between certificated value of SRM 2783 and our data, which is well within the acceptance range. Moreover, in order to verify the effect of sampling matrix, 19 pairs of collocated PM_2.5_ samples were synchronously collected on quartz fiber and Teflon^®^ membrane filters. The comparison results showed that there was a decent correlation between the Ni concentrations on the quartz fiber filters determined by our Epsilon 5 ED-XRF and those on Teflon^®^ membrane filters analyzed by the same technique (*R*^*2*^ = 0.920, *P* < 0.0001).

Eight PM_2.5_ samples (in a large range of PM_2.5_ mass loadings) were selected to compare the results generated from our ED-XRF against the inductively coupled plasma-atomic emission spectrometry (ICP-AES) conducted in the Environmental Chemistry Laboratory of Xi’an Jiaotong University. The good correlation observed between these two methods (*R*^*2*^ = 0.959, *P* < 0.0001) ensures the data quality of our results. In addition, replicate analyses on one sample loaded (five times) yielded an analytical precision of 14.9%. Laboratory blanks of the quartz fiber filters were analyzed to evaluate analytical bias, and the MDL for Ni was calculated to be 0.08 ng m^−3^. Moreover, replicable analyses were conducted for every eight samples; this was done to ensure that the stability and reproducibility of the instrument. The details of experimental description for the ED-XRF measurements are shown in Xu *et al*.^49^.

### Enrichment factor

Enrichment factor (EF) was calculated relatively to a reference from the earth’s upper continental crust, such as Fe, Si, Ti, or Al^51^. EFs have hence been used to evaluate crustal versus non-crustal contributions to elemental aerosol loadings. We used Fe as the normalizing element in this study^49^,^50^,^51^. The EFs were calculated following the equation (1)^52^:


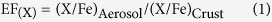


where X denotes any element of interest (Ni in this study). “Aerosol” in the numerator refers to the concentrations of Ni and Fe measured in the aerosol samples, while “Crust” in the denominator refers to the elemental concentrations in the upper crust^53^. If EF is approaching unity, the crust is probably the predominant source for Ni.

### Health risk assessment model

PM_2.5_ causes health risks to residents mainly in three ways: ingestion, inhalation, and dermal contact^1^. Inhalation is an important route of exposure to Ni in PM^3^. This study adopted health risk assessment models from the United States Environmental Protection Agency (USEPA) to evaluate inhalational health risks of Ni in Xi’an. The average exposure amount of Ni via the inhalation pathway per an individual’s body weight over a given time span for different age groups can be computed from the equation (2)^54^,^55^,^56^:


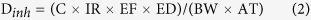


where D_*inh*_ represents average daily dose for non-cancer risk or lifetime average daily dose for cancer risk (mg kg^−1^ day^−1^). C represents Ni concentration in PM_2.5_ (mg m^−3^), and its upper limit of the 95% CI for the average is calculated from the SPSS software (Model 17.0). IR represents the volume of air that a person inhaled each day (m^3^ day^−1^). EF represents exposure frequency (day year^−1^). ED represents exposure duration (year). BW represents body weight (kg). AT represents the averaging time (days). The exposure parameters for each age group are shown in Table S4 in SI^57^. After D_*inh*_ is calculated, a hazard quotient (HQ) for non-cancer risk can be obtained by equation (3)^54^:


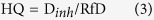


where the reference dose (RfD) (mg kg^−1^ day^−1^) is estimated as the maximum permissible risk on human by daily exposure. RfD value of Ni is 2 × 10^−5 ^57. The threshold value of RfD indicates whether there is adverse health effect during a lifetime. Then, hazard index (HI) can be obtained by summing up the individual HQ to estimate the total risks of all elements considered. If the HQ < 1, then non-carcinogenic effect is impossible; HQ ≥ 1, adverse health effect might likely appear^58^. In this study, since we only focused on the single element Ni, we compared Ni HQ (HQ = HI in this study) to unit in order to evaluate the non-cancer risk.

The cancer risk-incremental lifetime cancer risk (ILCR) can be calculated by multiplying the cancer slope factor (SF) of Ni with D_*inh*_ as equation (4)^54^,^55^,^56^:


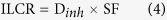


where the cancer SF of Ni is 0.84 ([mg kg^−1^ day^−1^]^−1^)^57^. SF value used in this study was not perfectly specific to PM_2.5_ Ni, potentially resulting in overestimation of health risks in the ambient aerosols, but reasonably equivalent to actual case^57^. For cancer risk, the value of 10^−6^ is an internationally accepted precautionary or threshold value, above which the risk is unacceptable^55^.

## Additional Information

**How to cite this article**: Xu, H. *et al*. A 10-year observation of PM_2.5_-bound nickel in Xi’an, China: Effects of source control on its trend and associated health risks. *Sci. Rep.* **7**, 41132; doi: 10.1038/srep41132 (2017).

**Publisher's note:** Springer Nature remains neutral with regard to jurisdictional claims in published maps and institutional affiliations.

## Supplementary Material

Supplementary Information

## Figures and Tables

**Table 1:** A statistical summary of PM_2.5_ and Ni concentrations over ten years (2004–2013).

Year	PM_2.5_ Mass (μg m^−3^)			Ni (ng m^−3^)			Ni/PM_2.5_^b^ (‰)
	Average	Stdev^a^	Range	Average	Stdev^a^	Range	
2004	169.5	101.3	28.9–631.4	6.5	5.0	0.44–53.8	0.038
2005	189.3	119.7	34.1–761.9	6.6	5.3	MDL^c^-32.8	0.035
2006	197.8	107.7	31.9–778.8	7.3	6.6	MDL-88.1	0.037
2007	190.9	102.2	30.3–617.2	7.7	8.3	0.76–76.6	0.040
2008	172.2	110.1	7.7–703.4	6.2	5.2	0.90–56.6	0.036
2009	162.0	100.0	14.2–635.3	7.0	7.1	MDL^c^-59.4	0.043
2010	149.8	89.0	7.6–597.7	5.8	5.1	1.20–41.9	0.039
2011	145.7	76.4	23.0–498.7	4.8	4.2	0.43–59.5	0.033
2012	160.5	105.5	18.7–766.2	4.2	5.4	0.13–70.9	0.026
2013	151.8	100.1	20.5–737.6	3.0	2.6	0.44–19.6	0.020

^a^Stdev: standard deviation.

^b^Ni/PM_2.5_ (‰): Ni concentration versus PM_2.5_ mass concentration (‰).

^c^MDL: Minimum Detection Limit of Ni (0.08 ng m^−3^).

**Table 2:** Annual variations of Ni enrichment factors (EFs) from 2004 to 2013 (using Fe as the normalizing element).

Year	Average	Stdev^a^	Spring	Summer	Autumn	Winter
2004	8.7	2.4	5.7	11.4	8.1	9.6
2005	10.2	4.6	6.9	16.2	8.1	6.4
2006	7.2	0.9	6.7	7.3	8.4	6.4
2007	8.9	4.7	2.8	10.7	8.0	8.1
2008	6.9	2.8	2.9	7.1	8.9	8.6
2009	8.5	3.7	7.7	13.8	5.4	7.1
2010	4.4	1.7	2.3	6.1	3.9	5.2
2011	8.7	3.3	3.8	9.4	9.7	10.7
2012	6.0	1.7	4.6	8.4	5.0	5.9
2013	3.8	1.3	2.0	4.8	3.8	4.6
Average	7.3	2.7	4.5	9.5	6.9	7.3

^a^Stdev: standard deviation.
