# Supplementary material for: A 10-year observation of PM2.5-bound nickel in Xi’an, China: Effects of source control on its trend and associated health risks
Source: Sci Rep. 2017 Jan 24;7:41132. doi: 10.1038/srep41132 (PMC5259713; doi:10.1038/srep41132)
Supplement: Supplementary Information [file srep41132-s1.doc]

**A 10-year observation of PM_2.5_-bound nickel in Xi’an, China: Effects of source control on its trend and associated health risks**

Hongmei Xu^1*,2,3^, Steven Sai Hang Ho^2,4^, Junji Cao^2*,5^, Benjamin Guinot^3^, Haidong Kan^6^, Zhenxing Shen^1^, Kin Fai Ho^7^, Suixin Liu^2^, Zhuzi Zhao^2^, Jianjun Li^2^, Ningning Zhang^2^, Chongshu Zhu^2^, Qian Zhang^1^ & Rujin Huang^2^

**Supplementary Information**

**Supplemental Figure Legends:**

**Figure S1.** Time series of daily PM_2.5_ Ni concentrations in Xi’an during 10 years.

**Figure S2.** Comparison of Ni concentrations among 2007, 2008 and 2009: daily (upper) and monthly variations (lower).

**Figure S3.** A: Raw coal consumption of industrial enterprises above designated size in Xi’an and coal percentage of total energy consumption; B: Investment completed in treatment of waste gas projects in Xi’an and newly increased disposal capacity of treatment of waste gas projects completed in Xi’an.

**Figure S4.** Seasonal variations of PM_2.5_ Ni concentrations in Xi’an from 2004 to 2013 (upper); Box chart of Ni concentration seasonal variations during 2004 to 2013 (The box plots indicated the seasonal average concentration and the min, 1^st^, 25^th^, 50^th^, 75^th^, 99^th^, and max percentiles. A normal curve is fitted to the measurements. Moreover, spring: March-May; summer: June-August; autumn: September-October, and winter: November-February) (lower).

**Figure S5.** Monthly variations of PM_2.5_ Ni concentrations in Xi’an from 2004 to 2013 (upper); Box chart of Ni concentration monthly variations during 2004 to 2013 (The box plots indicated the monthly average concentrations and the min, 1^st^, 25^th^, 50^th^, 75^th^, 99^th^, and max percentiles) (lower).

**Figure S6.** Monthly variations of enrichment factor of Ni in Xi’an from 2004 to 2013.

**Figure S7.** The probability (left) and cumulative probability (right) of cancer risk (ILCR) of Ni based on Monte Carlo simulation during the (a) workday and (b) non-workday periods.

**Table S1.** Energy consumption (raw coal, crude oil, gasoline, and diesel) of industrial enterprises above designated size and possession of civil vehicles in Xi’an.

| Year | Raw coal (ton) | Crude oil (ton) | Gasoline (ton) | Diesel (ton) | Civil vehicles (unit) |
| --- | --- | --- | --- | --- | --- |
| 2004 | 5175451 | 1411520 | 14238 | 45090 | 512802 |
| 2005 | 5572880 | 1717469 | 15987 | 41382 | 544586 |
| 2006 | 6251715 | 1663181 | 23354 | 49839 | 608155 |
| 2007 | 6805864 | 1772776 | 22396 | 42026 | 840376 |
| 2008 | 6656448 | 1556612 | 25712 | 47089 | 875005 |
| 2009 | 7840721 | 1688958 | 357367 | 363707 | 1012937 |
| 2010 | 7774450 | 1741676 | 30004 | 55290 | 1253461 |
| 2011 | 7548194 | 1531686 | 26419 | 53353 | 1445811 |
| 2012 | 7512882 | 2174699 | 24323 | 54390 | 1633257 |
| 2013 | 10311449 | 2131055 | 33086 | 66547 | 1862063 |

**Table S2. The statutory holidays (non-workday) in China during 2004-2013.**

| Statutory holiday | Specification | Applicable scope |
| --- | --- | --- |
| New Year’s Day | 1 day off, combined with the weekend except that the holiday is on Wednesday | 2004-2013 |
| Spring Festival | 3 days off, combined with the weekend | 2004-2013 |
| Tomb-sweeping Day | 1 day off, combined with the weekend except that the holiday is on Wednesday | 2008-2013 |
| Labor Day | 3 days off, combined with the weekend | 2004-2007 |
|  | 1 day off, combined with the weekend except that the holiday is on Wednesday | 2008-2013 |
| Dragon Boat Festival | 1 day off, combined with the weekend except that the holiday is on Wednesday | 2008-2013 |
| Mid-autumn Festival | 1 day off, combined with the weekend except that the holiday is on Wednesday, or combined with National Day | 2008-2013 |
| National Day | 3 days off, combined with the weekend | 2004-2013 |
| Weekend | 2 days off (Saturday and Sunday) except for the fore-mentioned holiday | 2004-2013 |

**Table S3. Meteorological factors in urban Xi’an from 2004 to 2013.**

| Year | Average temperature (℃) | Sunshine time (hour) | Raining days (day) | Annual precipitation (mm) | Average wind speed (m s^-1^) |
| --- | --- | --- | --- | --- | --- |
| 2004 | 15.2 | 1920.8 | 84 | 500.1 | 1.7 |
| 2005 | 14.9 | 1949.4 | 110 | 541.4 | 1.0 |
| 2006 | 15.8 | 2022.7 | 108 | 561.6 | 1.6 |
| 2007 | 15.6 | 1893.6 | 106 | 698.5 | 1.6 |
| 2008 | 14.9 | 1803.7 | 113 | 525.1 | 1.6 |
| 2009 | 15.1 | 1729.0 | 111 | 660.3 | 1.5 |
| 2010 | 15.3 | 1847.6 | 79 | 504.4 | 1.4 |
| 2011 | 14.5 | 1594.1 | 101 | 723.6 | 1.2 |
| 2012 | 14.6 | 1546.6 | 105 | 426.7 | 1.2 |
| 2013 | 15.8 | 2190.5 | 82 | 423.9 | 2.3 |
| Average | 15.2 | 1849.8 | 100 | 556.6 | 1.5 |

**Table S4**. Personal exposure parameters used in this study.

| Age group | IR | EF | ED | BW | AT (non-cancer) | AT (cancer) |
| --- | --- | --- | --- | --- | --- | --- |
| year old | m^3^ day^-1^ | day year^-1^ | year | kg | days | days |
| <1 | 12.8 | 280 | 1 | 7.8 | 1 × 365 | 70 × 365 |
| 1~3 | 13.7 | 259 | 3 | 12.3 | 3 × 365 | 70 × 365 |
| 3~6 | 13.8 | 243 | 3 | 17.6 | 3 × 365 | 70 × 365 |
| 6~11 | 16.6 | 266 | 5 | 25.3 | 5 × 365 | 70 × 365 |
| 11~16 | 21.9 | 255 | 5 | 42.3 | 5 × 365 | 70 × 365 |
| 16~21 | 24.6 | 211 | 5 | 55.4 | 5 × 365 | 70 × 365 |
| 21~71 | 19.7 | 269 | 50 | 58.6 | 50 × 365 | 70 × 365 |
| All ages | 17.6 | 255 | 70 | 60.0 | 70 × 365 | 70 × 365 |

**Figure S1.**

**Figure S2.**

**Figure S3.**

**Figure S4.**

**Figure S5.**

**Figure S6.**

**Figure S7.**
